# Antimicrobial Peptides: Novel Source and Biological Function With a Special Focus on Entomopathogenic Nematode/Bacterium Symbiotic Complex

**DOI:** 10.3389/fmicb.2021.555022

**Published:** 2021-07-14

**Authors:** Surajit De Mandal, Amrita Kumari Panda, Chandran Murugan, Xiaoxia Xu, Nachimuthu Senthil Kumar, Fengliang Jin

**Affiliations:** ^1^Laboratory of Bio-Pesticide Creation and Application of Guangdong Province, College of Agriculture, South China Agricultural University, Guangzhou, China; ^2^Department of Biotechnology, Sarguja University, Ambikapur, India; ^3^SRM Research Institute, SRM Institute of Science and Technology, Kattankulathur, India; ^4^Department of Biotechnology, Mizoram University, Aizawl, India

**Keywords:** antimicrobial peptides, multidrug-resistant pathogens, insects, nematodes, marine

## Abstract

The rapid emergence of multidrug resistant microorganisms has become one of the most critical threats to public health. A decrease in the effectiveness of available antibiotics has led to the failure of infection control, resulting in a high risk of death. Among several alternatives, antimicrobial peptides (AMPs) serve as potential alternatives to antibiotics to resolve the emergence and spread of multidrug-resistant pathogens. These small proteins exhibit potent antimicrobial activity and are also an essential component of the immune system. Although several AMPs have been reported and characterized, studies associated with their potential medical applications are limited. This review highlights the novel sources of AMPs with high antimicrobial activities, including the entomopathogenic nematode/bacterium (EPN/EPB) symbiotic complex. Additionally, the AMPs derived from insects, nematodes, and marine organisms and the design of peptidomimetic antimicrobial agents that can complement the defects of therapeutic peptides have been used as a template.

## Introduction

Antimicrobial peptides (AMPs) are small molecules that generally consist of 10–50 amino acids and are highly conserved in a wide range of species, including insects, nematodes, microbes, and mammals. AMPs serve as an essential component of the body’s immune system and defend against exogenous pathogens. They possess significant structural variations in the α-helices, β-strands with one or more disulfide bridges, loop, and extended structures associated with their broad-spectrum activities (Hancock, 2001; Pushpanathan et al., 2013). Other important factors associated with the functional activities of AMPs are size, hydrophobicity, charge, amphipathic stereo-geometry, and peptide self-association to the biological membrane (Nissen-Meyer and Nes, 1997; Marcos and Gandía, 2009; Pushpanathan et al., 2013). AMPs can be considered potential drug candidates to treat pathogenic microorganisms due to their broad-spectrum activity, lesser toxicity, decreased resistance development by the target cells, and capability to modulate the host immune response (Hancock and Patrzykat, 2002; Xu et al., 2019). AMPs can ameliorate the drug-resistant crisis and associated toxicity with conventional AMP drugs and also can be employed as an alternative to antibiotics (Lewies et al., 2019). They exhibit several similarities to antibiotics, such as killing microbial cells and targeting a broad spectrum of pathogens, including antibiotic resistance.

**FIGURE 1:**
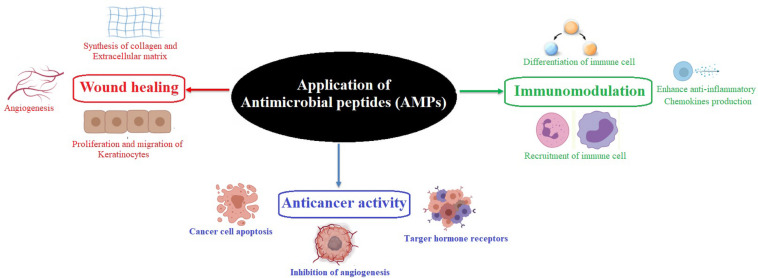
Role of an antimicrobial peptide in immunomodulation, wound healing, and anticancer activity.

**FIGURE 2:**
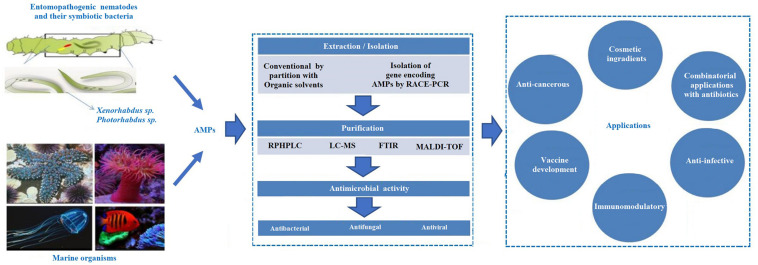
Schematic representation of the antimicrobial peptides (AMPs) extracted from the different source and their applications.

Moreover, compared to antibiotics, AMPs have unique epitopes that serve as protease recognition sites, thereby less likely to be targeted by the protease (Zasloff, 2002; Lai and Gallo, 2009). Different mechanisms, such as inhibition of gene expression or protein synthesis, inhibition of cell wall synthesis, or delocalization of bacterial cell surface proteins are commonly employed by the AMPs (Baltzer and Brown, 2011). Most of the AMPs are cationic and capable of adapting to amphipathic conformations. This helps them interact with the negatively charged bacterial cell wall and integrate it into the lipid bilayers (Haney et al., 2017; Zharkova et al., 2019). The success of AMPs against multidrug-resistant pathogens is due to the widescale multitargeted action (Zharkova et al., 2019). They are also active at lower minimum inhibitory concentrations (MICs) as compared to antibiotics. AMPs demonstrate higher killing effects and show a narrower mutation-selection window, accounting for the less likely development of resistance to AMP (Fantner et al., 2010; Yu et al., 2018). They are also active against biofilm-producing antibiotic-resistant microbes and induce non-opsonic phagocytosis. However, the combined use of AMPs with other antimicrobial compounds such as specific antibiotics may play a vital role against multidrug-resistant pathogens and associated adverse health conditions. In addition, some AMPs have been identified to exhibit antiviral activities (Chia et al., 2010; Van Der Does et al., 2010; Chung and Kocks, 2011; Steckbeck et al., 2014; Elnagdy and AlKhazindar, 2020). The AMPs play an essential role in modulating immunogenic activities, improving wound healing, enhancing chemokine production, exhibiting anti-inflammatory properties, regulating epithelial cell differentiation, and modulating angiogenesis (Koczulla and Bals, 2007; Mahlapuu et al., 2016; Otvos, 2016; Patrulea et al., 2020; Figure 1). Nowadays, scientists are participating in developing enhanced AMPs with novel modes of actions to replace or complement traditional antibiotics to treat various diseases (Morikawa et al., 1992; Wang et al., 2016). So far, 3257 AMPs have been reported from six kingdoms (bacteria, archaea, fungi, protists, plants, and animals) in the Antimicrobial Peptide Database^1^ (Wang et al., 2016). This review provides insights into developing different AMPs from novel sources and their multifunctional properties and elaborates their future prospects (Figure 2). Particular focus has been given to the AMPs in bacteria that form a symbiotic relationship with the entomopathogenic nematodes (EPNs), displaying varied modes of actions.

## AMPs in Insects

Insects represent the largest class in the animal kingdom and are found in most of the biological niches. One of the critical features of their successful adaptation is their resistance to various pathogens. The AMPs play a critical role in innate immunity against insect pathogens (Bulet et al., 1999). They produce a large number of AMPs that varies between species, ranging from 50 (*Harmonia axyridis*) to 0 (*Hermetia illucens*) (Gerardo et al., 2010; Vilcinskas, 2013; Vogel et al., 2018). Cecropin, the first insect AMP, was isolated and characterized from *Hyalophora cecropia* (Steiner et al., 1981). Since then, many insect AMPs have been reported, which are mainly classified into three groups based on the sequence and structural features, i.e., linear peptides with α-helices that lack cysteine residues and cyclic peptides containing cysteine residues and peptides with an overexpression of proline and glycine residues (Hetru, 1998; Bulet et al., 1999). The most explored insect AMPs are defensin, cecropin, drosocin, attacin, diptericin, ponericin, drosomycin, and metchnikowin. However, it is expected that insects may have more AMPs with novel modes of action (Mylonakis et al., 2016).

Cecropins are small peptides that destroy bacterial cell membranes, inhibit proline uptake, and cause leaky membranes (Moore et al., 1996). It has also been reported that cecropin A (CecA) destroys uropathogenic *Escherichia coli* (UPEC) cells, alone or in combination with nalidixic acid (NAL), and could be a practical approach to treat antibiotic-resistant UPEC infections (Kalsy et al., 2020). CecA from *H. cecropia* exhibits only antibacterial activity, whereas CecA from *Anopheles gambiae* exhibits antibacterial and antifungal activities (Bulet et al., 2004). BR003-CecA from *Aedes aegypti* actively inhibits multiple species of Gram-negative bacteria (GNB), including *A. baumannii* (Jayamani et al., 2015). Cec D from *Galleria mellonella* exhibits vigorous activity against Gram-positive bacterium (GPB) *Listeria monocytogenes* (Mukherjee et al., 2011). Defensins are the second primary class of inducible insect AMPs active against GPB, including *Staphylococcus aureus*, but are less active against GNB (Hetru et al., 2003; Gomes and Fernandes, 2010). Few defensins also possess antifungal activities against filamentous fungi, e.g., gallerimycin from the greater wax moth *G. mellonella* (Langen et al., 2006). Insect defensin-like peptides are found in *Leiurus quinquestriatus* and *Androctonus australis* (Cociancich et al., 1993; Ehret-Sabatier et al., 1996). Defensin-like peptide 4 (DLP4) reported from the black soldier fly is active against GPB (Park et al., 2015).

The AMP drosocin, isolated from *Drosophila melanogaster*, is a 19-residue peptide containing six proline and four arginine residues (McManus et al., 1999). Glycosylated drosocin is active against *E. coli* and fungi (Imler and Bulet, 2005). These O-glycosylated AMPs are also found in other insects such as *Pyrrhocoris apterus* (pyrrhocoricin), *Bombyx mori* (lebocins), and *Myrmecia gulosa* (formations) (Cociancich et al., 1994; Hara and Yamakawa, 1995; Mackintosh et al., 1998; Wu et al., 2018).

Attacins, glycine-rich AMP, were first discovered in *H. cecropia* and is active against GNB (Hultmark et al., 1983; Carlsson et al., 1991). Attacins from *Spodoptera exigua* exhibit activity against *E. coli*, *Pseudomonas cichorii*, *Bacillus subtilis*, *L. monocytogenes*, *Trypanosoma brucei*, *Citrobacter freundii*, and *Candida albicans* (Hu and Aksoy, 2005; Kwon et al., 2008; Bang et al., 2012). Attacins and attacin-related proteins are also isolated from *B. mori*, *Heliothis virescens*, *Trichoplusia ni*, *Samia cynthia ricini*, and *Musca domestica* (Dushay et al., 2000; Geng et al., 2004).

Diptericin (9 kDa), found in *D. melanogaster*, *Sarcophaga peregrina*, and *Mayetiola destructor*, is active against GNB such as *E. coli*, *Erwinia herbicola* T, and *E. carotovora* (Keppi et al., 1989; Ishikawa et al., 1992; Reichhart et al., 1992).

However, limited reports are available on antifungal peptides in insects such as drosomycin from *D. melanogaster*, termicin from termites, heliomicin from *H. virescens*, and gallerimycin peptide from *G. mellonella* (Fehlbaum et al., 1994; Da Silva et al., 2003; Schuhmann et al., 2003). The antifungal peptide drosomycin is active against fungal pathogens, whereas thanatin is effective against a broad range of β-lactamase-producing *E. coli* (Bulet et al., 1999; Hou et al., 2011).

Xu et al. (2019) reported a novel Moricin (Px-Mor) from the diamondback moth that showed a broad-spectrum activity against GPB, GNB, and fungi, including the opportunistic human pathogen *Aureobasidium pullulans*. They suggested that Px-Mor can be used as a potential topical antimicrobial agent (Xu et al., 2019). These results indicate the importance of insect-derived AMPs against pathogens and could be further employed against multidrug-resistant pathogens or in combination with existing antibiotics (Table 1).

**TABLE 1:** Recently identified insect AMPs with their mechanism of action.

**Name of AMP**	**Type of AMP**	**Source**	**3D structure**	**Pathogenic species**	**Molecular mechanism**	**Inhibitory concentration**	**References**
ETD151 (Heliomicin)	Defensin	*Heliothis virescens*	Combine helix and beta structure	*Botrytis cinerea*	Interact with glucosylceramides of the fungal membrane	IC_50_ = 0.59 μM	Aumer et al., 2020
Holosins		*Ixodes holocyclus*	Cys-stabilized α/β-fold	*Staphylococcus aureus*, *Listeria grayi*, *F. graminearum*, and *C. albicans*	Peptide–lipid interactions result in the formation of membrane penetrating pores	MIC = 8 μM MIC = 5 μM	Cabezas-Cruz et al., 2019
Oxysterlins	Cecropin	*Oxysternon conspicillatum*	Linear α-helix	*Staphylococcus saprophyticus*, *Klebsiella pneumoniae*, and *Pseudomonas aeruginosa*	Membrane lysis due to formation of pores	MIC = 12.5 μg/ml MIC = 3.12 μg/ml MIC = 12.5 μg/ml	Toro Segovia et al., 2017
Cecropin D		*Galleria mellonella*	α-Helix	*K. pneumoniae* (MDR), *P. aeruginosa* (MDR)		MIC = 256 μg/ml MIC = 32 μg/ml	Ocampo-Ibáñez et al., 2020
Cecropin B		*Antheraea pernyi*		*P. aeruginosa*		MIC = 0.4 μg/ML	Wu et al., 2012; Yang et al., 2018; Gholizadeh and Moradi, 2020
Cecropin AD		*Hyalophora cecropia*		*Staphylococcus aureus*		MIC = 0.2 μg/ml	
HI-attacin	Attacin	*Hermetia illucens*	Unknown	*E. coli* and methicillin-resistant *Staphylococcus aureus*	Blocking the synthesis of the major outer membrane proteins, thus disturbing the integrity of the cell wall	NS	Shin and Park, 2019
Prolixicin		*Rhodnius prolixus*		*E. coli*, *Citrobacter freundii*, *Enterobacter aerogenes*, and *Bacillus coagulans*		MIC = 1.6 μM MIC = 12.5 μM	Ursic-Bedoya et al., 2011
SlLeb-1	Lebocin	*Spodoptera litura*	Rich	*E. coli* and *B. subtilis*	Disrupt cell membrane and cause cell elongation	MIC = 50 μM	Yang et al., 2018; Yang et al., 2020
Apidaecin IB	Drosocin	*Apis cerana*	Rich	*Escherichia coli* and *Klebsiella pneumoniae*	Binds to the substrate binding site of *E. coli* DnaK to compete with natural substrates	NS	Berthold and Hoffmann, 2014; Feng et al., 2020
Api795	Apidaecin			*P. aeruginosa*	Insert into bacterial mimic membranes and initiates a structural change leading to a thicker and more rigid membrane layer	MIC = 8 μg/ml	Bluhm et al., 2016
EtDip	Diptericin	*Eristalis tenax*	Unknown	*Candida albicans* FH2173 and *Mycobacterium smegmatis* ATCC 607		MIC > 1024 μg/ml MIC = 64 μg/ml	Hirsch et al., 2020
Mtk	Metchnikowin	*Drosophila melanogaster*	Rich	*Fusarium graminearum*	Interacts with the fungal enzyme β(1,3)-glucanosyltransferase Gel1 (*Fg*BGT), which is one of the enzymes responsible for fungal cell wall synthesis	NS	Moghaddam et al., 2017
Ponericin-Q42	Ponericins	*Ectatomma quadridens*	α-Helical folds	*Arthrobacter globiformis*, *B. subtilis*, *E. coli* MH1, and *P. aeruginosa* PAO1	Membrane blebbing, formation of swollen cells and finally membrane destruction and cell death	MIC = 0.2 μM MIC = 0.6 μM MIC = 10 μM	Pluzhnikov et al., 2014
Jelleine-I	Jelleines	*Apis mellifera*	Unknown	*Candida glabrata* and *C. albicans*	Increase the production of cellular ROS and bind with genome DNA	MIC and MFC = 30 μg/ml MIC and MFC = 61 μg/ml	Jia et al., 2018
Pyrrhocoricin	Pyrrhocoricin	*Pyrrhocoris apterus*	Non-helix beta	Cell-free *E. coli* system and *Cryptosporidium parvum*	Inhibit the protein folding activity of the ATP-dependent DnaK/DnaJ molecular chaperone system	IC for transcription = 427 μM	Boxell et al., 2008; Taniguchi et al., 2016
Melittin	Melittin	*Apis mellifera*	Helix	*Lactobacillus casei* and *Streptococcus mutans*	Interact with bacterial membrane	MIC = 4 μg/ml MIC = 40 μg/ml	Leandro et al., 2015

## AMPs in Nematode

Antimicrobial peptides are produced by microorganisms associated with insect symbioses and play a significant role in maintaining the symbiotic microbe in specific anatomical compartments (Ovchinnikova et al., 2004; Tasiemski et al., 2015). A lot of the literature have highlighted the EPN/entomopathogenic bacterium (EPB) symbioses. Nematodes are specialized organisms with the ability to adapt both free-living and parasitic lifestyle in different environments. Nematodes also serve as a novel invertebrate model to study innate immunity and host–pathogen interactions (Kurz and Tan, 2004; Nicholas and Hodgkin, 2004). EPNs and their associated bacteria have evolved with several defense mechanisms to elude and counteract the host (insect) immune responses (Brivio et al., 2018; Brivio and Mastore, 2020). The nematode *Steinernema carpocapsae* can produce proteolytic secretions that can interfere with the host immune system in *S. feltiae*, *S. glaseri*, and *G. mellonella* (Balasubramanian et al., 2010; Chaubey and Garg, 2019). Similarly, the surface proteins of *S. glaseri* protect from encapsulation by the host immune system of *Popillia japonica* (Wang and Gaugler, 1999; Chaubey and Garg, 2019). It has also been reported that symbiotic bacteria and nematode cooperate to overcome the host immune response. Many defense molecules are produced as immune effectors against various microbial infections (Bulet et al., 2004). The AMP cecropins are found in the worm *Ascaris suum* (cecropin-P1, cecropin-P2, cecropin-P3, and cecropin-P4), a parasite inhabiting the intestine of pigs. These short AMPs, rich in serine residues, are stabilized by the disulfide bonds and contain potential antimicrobial activities against BPB (*S. aureus*, *B. subtilis*, *Micrococcus luteus*) and GNB (*Pseudomonas aeruginosa*, *Salmonella typhimurium*, *Serratia marcescens*, and *E. coli*) and are less effective against fungi (*Saccharomyces cerevisiae*, *C. albicans*) (Andrä et al., 2001; Andersson et al., 2003; Pillai et al., 2005; Bruno et al., 2019). Cecropin P1-like sequences were also identified in two other species, i.e., *Ascaris lumbricoides* and *Toxocara canis* (Pillai et al., 2005).

Another group of AMPs called the caenopores belong to the saposin-like protein (SAPLIP) superfamily detected in *Caenorhabditis elegans*. It contains conserved positions of six cysteine residues. Caenopore-1 (SPP-1), caenopore-5 (SPP-5), and caenopore-12 (SPP-12) exhibit antimicrobial activity against *B. megaterium*, *E. coli*, and SPP-12 *Bacillus thuringiensis* (Roeder et al., 2010; Hoeckendorf et al., 2012).

Defensins are the most studied AMPs in nematodes. *Ascaris suum* antibacterial factors (ASABFs) was the first nematode defensin identified in *C. elegans*. They are short AMPs with eight cysteine residues that form four disulfide bonds except for ASABF-6Cys-α (Minaba et al., 2009; Tarr, 2012). These peptides are primarily active against GPB, especially the common pathogen *S. aureus*. However, it is less effective against GNB and yeast (Tarr, 2012). A recent study by Lim et al. (2016) reported two novel *C. elegans* AMPs (NLP-31 and Y43C5A.3) that exhibit antimicrobial activity against *Burkholderia pseudomallei*, the causative agent of melioidosis, by interfering with DNA synthesis. They also revealed that these AMPs might act by modulating host cytokine production to interfere with the inflammatory response, and modifications could enhance anti-*B. pseudomallei* activities (Lim et al., 2016).

## AMPs Linked With EPN/EPB Symbiotic Complex

Several bacterial genera belonging to the Enterobacteriaceae family are mutualistically associated with the EPNs (Boemare, 2002). These EPNs, with their symbiotic bacteria, are lethal to many soil insects, as they synthesize diverse secondary metabolites, including small AMPs. These nematode-associated microbes exist in two distinct phases: phase 1, where they are generally associated with the nematodes, and phase 2, where they may also colonize with the nematode. However, they have never been reported to be associated with the naturally occurring nematodes. Both the phases have distinguished physiological, biochemical, and behavioral features; also phase 1 is considered more virulent than phase 2 (Akhurst et al., 1990; Volgyi et al., 1998; Abdel-Razek, 2002; Sugar et al., 2012). During the infective juvenile (IJ) stage, the nematodes enter inside the insects by piercing the body wall or via natural openings and releasing these bacteria inside the hemocoel. They reproduce exponentially, producing bioactive compounds with broad-spectrum antimicrobial activities (Sanda et al., 2018). They provide nutrients to the nematodes and protect them from environmental predators such as bacteria and fungi. They also compete for nutrition with other microbes, including the saprophytic soil microbes and the bacteria present in the insect gut or cuticle of the nematode. The elimination of the competitors is facilitated by the production of colicin E3-type killer proteins, insect toxin complexes, phage-derived bacteriocins, and several secondary metabolites (Thaler et al., 1995; Ffrench-Constant and Waterfield, 2006; Singh and Banerjee, 2008; Bode, 2009; Piel, 2009). The presence of high content of non-ribosomal peptide synthetase (NRPS) and polyketide synthase (PKS) genes facilitates them to produce novel and new bioactive molecules (Tobias et al., 2017). These bioactive molecules disrupt the insect’s metabolic and functional properties, leading to septicemia (Khandelwal and Banerjee-Bhatnagar, 2003; Tran and Goodrich-Blair, 2009; Ellis and Kuehn, 2010; Brivio et al., 2018). Nematodes also play a significant role in the pathogenicity of the nemato-bacterial complex (Han and Ehlers, 2000; Chang et al., 2019). The EPNs, along with the mutualistic bacteria, kill their host within 48−72 h (Forst and Nealson, 1996). These features are now being exploited for the biological control of pests (Brivio and Mastore, 2018).

The bacterial genus *Xenorhabdus* is often found in close association with EPNs of the family Steinernematidae (Webster et al., 2002). *Xenorhabdus* synthesizes and releases antibiotic compounds in the host hemocoel that suppresses the microbial competitors, thereby manipulating the environment to promote growth, proliferation, and nematode development (Vallet-Gely et al., 2008; Richards and Goodrich-Blair, 2009; Gaugler, 2018). The antimicrobial compounds produced by these bacterial genera are highly toxic to the insect but not toxic to the nematodes. Various surface structures such as pili/fimbriae, flagella, and the outer membrane vesicles (OMVs) present in the *Xenorhabdus* interact with the host and promote adhesion and invasion of the host tissues. They also promote larvicidal activity by releasing proteases, lytic factors, and phospholipase C (Brivio et al., 2018). Ribosomal-encoded bacteriocins (xenorhabdicins) are found in *Xenorhabdus nematophilus*. These AMPs compete against more closely related bacteria, such as other *Xenorhabdus* and *Photorhabdus* strains (Thaler et al., 1995). The indole-containing Xenematide from *Xenorhabdus nematophila* exhibits moderate antibacterial and insecticidal activities (Lang et al., 2008). Two novel depsipeptides, xenematides F and G, were isolated from *Xenorhabdus budapestensis* SN84 with high antibacterial activity (Xi et al., 2019).

The cyclic peptide-antimicrobial-*Xenorhabdus* (PAX) lipopeptides, obtained by the fermentation of the *X. nematophila* F1 strain, exhibit significant activity against plants and human fungal pathogens and moderately effective against a few bacteria and yeast (Gualtieri et al., 2009). Two novel AMPs GP-19 and EP-20 from the bacterial strain *X. budapestensis* NMC-10. GP-19 exhibited inhibitory activity mainly against bacteria, while EP-20 was highly effective against plant pathogens. The synthetic GP-19 and EP-20 peptide exhibited inhibitory activities against the fungal pathogen *Verticillium dahlia* and *Phytophthora capsici* with EC_50_ values of 17.54 and 3.14 μg/ml, respectively (Xiao et al., 2012).

The AMPs xenocoumacin 1 (XCN 1) and 2 (XCN 2), from the bacterium *X. nematophila*, is effective against GPB and fungi. This peptide is synthesized by the PKS/NRPS multienzyme (xcnA-N) (McInerney et al., 1991; Reimer, 2013). Six novel linear peptides (rhabdopeptides) in *X. nematophila* and two other rhabdopeptide derivatives by *X. cabanillasii* were also identified (Reimer et al., 2013).

Nematophins, from *X. nematophila* YL001, inhibit mycelial growth of *Rhizoctonia solani* and *Phytophthora infestans* with an EC_50_ value of 40.00 and 51.25 μg/ml, respectively, and can be employed as a potential biopesticide in the agriculture sector (Zhang et al., 2019). Similarly, the novel peptide, xenoamicin, tridecadepsipeptides with hydrophobic amino acids, from the entomopathogenic *X. doucetiae* DSM 17909 and *X. mauleonii* DSM 17908 was effective against *Plasmodium falciparum* (Zhou et al., 2013).

Another dipeptide xenobactin was isolated from *Xenorhabdus* sp., strain PB30.3, and szentiamide from *X. szentirmaii*. Both AMPs are active against *P. falciparum* and have moderately effective against *T. brucei rhodesiense* and *Trypanosoma cruzi* (Nollmann et al., 2012; Grundmann et al., 2013). Similarly, the depsipentapeptide chaiyaphumine A from *Xenorhabdus* sp. PB61.4 was effective against *P. falciparum* (IC_50_ of 0.61 μM) and other protozoal tropical disease-causing agents (Grundmann et al., 2014). *Xenorhabdus indica* can produce depsipeptides and lipodepsipeptides with an additional fatty acid chain linked to one of the amino acids, also called taxlllaids (A–G), and exhibits antiprotozoal activity (Kronenwerth et al., 2014). Taken together, these reports suggest that the mutualistic association between *Xenorhabdus* and Steinernematidae could serve as a potential source for novel AMPs against bacteria, fungi, and protozoal disease-causing agents.

The bacterium *Photorhabdus* spp. forms a symbiotic relationship with the EPNs of the genus *Heterorhabditis* (Gerrard et al., 2006). They cause pathogenicity in most insects post invading the hemolymph (Boemare et al., 1997). Genomic analysis of Photorhabdus can interpret the relation between pathogenesis and symbiosis, thereby providing vital information for the development of biocontrol agents. The genomic sequence of *P. luminescens* subsp. *laumondii* strain TT01 revealed several genes that encode toxins, hemolysins, adhesins, hemolysins, proteases, lipases, and a wide array of antibiotics. Two identified protein, PirA and PirB, exhibit similarity to both δ-endotoxins (*B. thuringiensis*) and a developmentally regulated protein from a beetle (Leptinotarsa decemlineata) (Duchaud et al., 2003; Waterfield et al., 2005). Research on the larvicidal activity of *Photorhabdus* sp. showed that *Photorhabdus* insect-related (Pir) protein is associated with high toxicity against the primary vector of dengue virus *A. aegypti* and *Aedes albopictus* (Ahantarig et al., 2009). These novel insecticidal proteins could further be exploited to develop alternative agents to control insect pests. Genomic analysis of *P. luminescens* subsp. *laumondii* strain TT01 indicates the presence of several enzymes associated with the secondary metabolite biosynthesis. The genomic sequence analysis identifies biosynthetic gene clusters associated with the synthesis of linear or cyclized peptides, lipopeptides, or depsipeptides; NRPS; unusual fatty acid synthase or a FAS/PKS hybrid; and siderophore biosynthesis (Bode, 2009). *Photorhabdus* sp. also produces numerous antimicrobials such as isopropyl stilbene, ethylstilbenes, anthraquinones (AQs) photobactin, ethyl stilbene, epoxystilbene, and ulbactin E (Li et al., 1995; Webster et al., 2002; Bode, 2009). The bioactive compounds exhibit a broad range of antimicrobial activities. *Photorhabdus* antibacterial compounds include trans-stilbenes and anthraquinone pigments (Boemare and Akhurst, 2002) that have enthralled substantial interest in the agronomic and pharmaceutical sectors (Webster et al., 2002; Hazir et al., 2016). Phthalic acid or 1,2-benzene dicarboxylic acid purified from *Photorhabdus temperata* M1021 exhibits an antibacterial activity with MIC values of 0.1 and 0.5 M (Ullah et al., 2014), benzaldehyde exhibits an antibacterial activity with MIC values of 6 and 10 mM, and antifungal activity with MIC values between 8 and 10 mM (Ullah et al., 2015). *P. temperata* subsp. *temperata* inhibits the growth of 10 strains of drug-resistant bacteria (Muangpat et al., 2017), including *S. typhimurium* KCTC 1926 and *M. luteus* KACC 10488 (Jang et al., 2012). Therefore, *Photorhabdus* spp. can be a suitable biocontrol agent in drug industries. AMPs from nemato-bacterial complexes with their inhibitory concentrations are shown in Table 2.

**TABLE 2:** Antimicrobial peptides from nematobacterial complexes with their inhibitory concentrations.

**Name of AMP**	**Source**	**Pathogenic species**	**Inhibitory concentration**	**References**
Xenematide C	*Xenorhabdus budapestensis* SN19	*Botrytis cinerea*	EC_50_ = 22.71 μg/ml	Xing-zhong et al., 2016
Xenematides F	*Xenorhabdus budapestensis* SN84	*P. aeruginosa*	MIC = 32 μg/ml	Xi et al., 2019
Xenematides G		*B. subtilis*	MIC = 16 μg/ml	
PAX lipopeptides Xenocoumacin 2	*Xenorhabdus khoisanae* SB10	*B. subtilis* subsp. *subtilis Escherichia coli Candida albicans*	NS	Dreyer et al., 2019
Diketopiperazines	EPN *Rhabditis* sp.	*Penicillium expansum*	MIC = 2 μg/ml	Kumar et al., 2013
Nematophin	*Xenorhabdus nematophila* YL001	*Rhizoctonia solani Phytophthora infestans*	EC_50_ = 40.00 μg/ml EC_50_ = 51.25 μg/ml	Zhang et al., 2019
Nematophin	*Xenorhabdus* PB62.4	*Staphylococcus aureus*	MIC = 0.7 μg/ml	Cai et al., 2017
GP-19 EP-20	*Xenorhabdus budapestensis* NMC-10	*Verticillium dahlia Phytophthora capsici*	EC_50_ = 17.54 μg/ml EC_50_ = 3.14 μg/ml	Xiao et al., 2012
Threonine–glutamine dipeptide) domain containing protein	*Bacillus cereus*	*E. coli*, *S. aureus*, and *B. subtilis*	MIC = 62.55 μg/ml MIC = 125 μg/ml MIC = 250 μg/ml	Anju et al., 2015
Xenocoumacin 1 Xenocoumacin 2	*Xenorhabdus nematophila*	*Botrytis cinerea*	Inhibition rate of 100 ml/L cell-free filtrate on the mycelial growth of the pathogens is 100%	Guo et al., 2017
Nematophins, Xenocoumacins and Xenorhabdins	*Xenorhabdus* assam-isolate (SG as1)	*Macrophomina phaseolina*	EC_50_ = 55.98 μg/ml	Sharma et al., 2016
Cabanillasin	*Xenorhabdus cabanillasii*	*Fusarium oxysporum*	IC_50_ = 6.25 μg/ml	Houard et al., 2013
Xenobactin	*Xenorhabdus* sp. PB30.3	*Micrococcus luteus Plasmodium falciparum* NF 54 *Trypanosoma brucei rhodesiense* STIB900 *Trypanosoma cruzi* Tulahuen C4	MIC = 64 μg/ml IC_50_ = 12.45 μg/ml IC_50_ = 12.45 μg/ml IC_50_ = 67.03 μg/ml	Grundmann et al., 2013
Xenortide D	*Xenorhabdus nematophila*	*Plasmodium falciparum Trypanosoma brucei*	NS	Reimer et al., 2014
Taxlllaids	*Xenorhabdus indica* (DSM 17382)	*Plasmodium falciparum*	NS	Kronenwerth et al., 2014
Phototemtide A	*Photorhabdus temperata* Meg1	*Plasmodium falciparum Trypanosoma brucei rhodesiense*	IC_50_ = 9.8 μM IC_50_ = 62 μM	Zhao L. et al., 2020

## The Targets and Mechanism of Action of AMPs Derived From Nematobacterial Complexes

**FIGURE 3:**
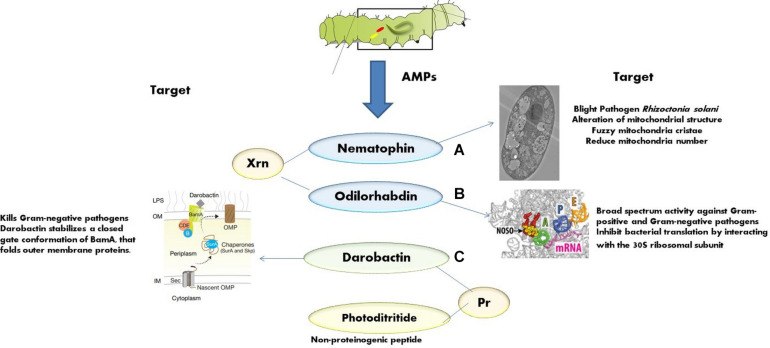
Mode of action of various AMPs: **(A)** nematophins act on mitochondrial structure of *Rhizoctonia solani* [adapted from Zhang et al. (2019)]; **(B)** odilorhabdins act on the ribosome and inhibit protein synthesis in Gram-negative bacteria [adapted from Pantel et al. (2018)]; **(C)** darobactins act on outer membrane chaperone [adapted from Imai et al. (2019)].

The AMPs from *Xenorhabdus* spp. and *Photorhabdus* spp. are non-lethal to nematode but toxic to insect pathogens and other opportunistic microorganisms with unique targets and modes of action. This section highlights some of the recently identified AMPs from EPB with novel modes of action, namely, nematophin, odilorhabdin, darobactin, and photoditritide (Figure 3).

### Nematophin

First isolated from *X. nematophilus* strain BC1 (Li et al., 1997), it contains 3-indoleethyl (3′-methyl-2′-oxo) pentanamide with an N-terminal α-keto group and a C-terminal tryptamine residue. Recently, few novel nematophin analogs were identified from *Xenorhabdus* strains (Cai et al., 2017). Nematophin is effective against *S. aureus* (MIC = 0.125 μg/ml) (Li et al., 1997), methicillin-resistant *S. aureus* (MRSA) (MIC = 1.5 μg/ml), and fungal pathogens, *Botrytis cinerea* (MIC = 12 μg/ml) (Li et al., 1997) and *R. solani* (MIC = 40 μg/ml) (Zhang et al., 2019). The synthetic nematophin analog with *N*-methyl substitution exhibits nanomolar activity toward *S. aureus* (15 ng/ml), *S. intermedius* 9503 (50 ng/ml) (Himmler et al., 1998), *S. hyicus* (60 ng/ml), MRSA ATCC 43300 (31 ng/ml), and methicillin-susceptible *S. aureus* ATCC 29213 (125 ng/ml) (Wesche et al., 2019). Recent studies indicate that nematophin is a potent biopesticide against a necrotrophic fungal pathogen *R. solani*. It interferes with the sclerotial development and hyphal morphology of *R. solani* at 40.00 μg/ml and germination at 15.00 μg/ml. The ultrastructure shows that the hyphae becomes twisted, shriveled, and deformed at the growing points after the exposure to nematophin at 40.00 μg/ml, and the mitochondrial structural abnormalities such as reduction in number, vacuolar degeneration, and fuzzy cristae are also observed (Figure 3A).

### Odilorhabdin

This is a new class of AMP with broad-spectrum activity encoded by the enzymes (of NRPS gene cluster) of *X. nematophila*. This peptide binds to the decoding center of the small ribosomal subunit, leading to faulty coding procedure and prohibits non-cognate aminoacyl-tRNAs binding (Pantel et al., 2018). Odilorhabdin can directly bind with the new site on 16S rRNA (Figure 3B) and with the anticodon loop of the A-site aminoacyl-tRNA concurrently, resulting in the precision of translation decreased. At very high concentrations, odilorhabdin hinders the ribosome movement on mRNA (Pantel et al., 2018). Studies reported that odilorhabdin acts against Gram-negative and Gram-positive bacterial pathogens, including carbapenem-resistant Enterobacteriaceae, which strain especially shows resistance toward many classes of available antibiotics and causes severe infections with a 50% mortality rate (van Duin et al., 2013).

### Darobactin

It is a novel peptide antibiotic produced by *Photorhabdus khanii* HGB1456 (Imai et al., 2019) that is effective against several Gram-negative drug-resistant pathogens. Instead of targeting the enzymes, darobactin targets outer membrane chaperone BamA (Figure 3C), catalyzing the insertion and folding of β-barrel outer membrane proteins in many Gram-negative pathogens. As the target of darobactin is a cell surface protein, there is no permeability obstacle encountered (Imai et al., 2019). No antibiotics were reported to act on the two surface proteins, namely, BamA and LptD, present on the GNB; therefore, darobactin could act as a potential drug candidate due to its distinctive sizeable molecular structure fused rings and unusual cell surface target (Konovalova et al., 2017).

### Photoditritide

Photoditritide is the first non-proteinogenic peptide reported from *P. temperata* Meg1 through promoter exchange (Maglangit et al., 2021). Photoditritide 19 consists of two tyrosines, two homo-arginines, and two tryptophans (Bode et al., 2015). It is effective against *E. coli* (MIC = 24 μM), *M. luteus* (MIC = 3.0 μM), and antiprotozoal activity against *P. falciparum* (IC_50_ = 27 μM), *T. cruzi* (IC_50_ = 71 μM), and *T. brucei rhodesiense* (IC_50_ = 13 μM) (Bode et al., 2015).

The increasing evidence of antibiotic resistance is a serious issue. Drug-resistant pathogens develop new resistance mechanisms and interfere in the treatment of common infections. Moreover, multidrug resistance pathogenic strains have developed tolerance against most of the available antibiotics. Researchers searching for novel sources of antimicrobial agents through synthetic compound library screening have mostly failed to get efficient antimicrobial agents (Payne et al., 2007). Therefore, exploiting new natural antimicrobial sources to fill the research gap in antimicrobial drug discovery is a promising approach. Most of the antibiotics used to date belong to soil actinomycetes. The present review aims to compile novel natural sources, highlighting the unnoticed and ignored sources to identify new AMPs with a unique mode of action. The marine ecosystem presents a vast repository of microorganisms, invertebrates, and vertebrates that produce various natural products and AMPs with the perspective of treating several infectious diseases (Bertrand and Munoz-Garay, 2019).

## Marine-Derived Antimicrobial Peptides

The marine ecosystem encompasses an unprecedented variety of organisms that have shown remarkable contribution in discovering and developing novel biomolecules, nutraceuticals, and secondary metabolites that pave the way to produce antimicrobial agents (Malve, 2016; Sekurova et al., 2019; Figure 1). AMPs derived from marine sources are novel and revolutionary therapeutic agents with distinctive pharmacological properties such as antimicrobial, antiproliferative, antioxidant, anticoagulant, antihypertensive, antidiabetic, and antiobesity properties (Jo et al., 2017).

### Antimicrobial Peptides Derived From Marine Invertebrates

Marine invertebrates produce AMPs to activate innate immune machinery to recognize, neutralize, and eliminate invading pathogens (Loker et al., 2004). A wide variety of corals produce structurally unique bioactive metabolites that can serve as significant novel compounds in drug development against various human diseases. For example, the marine fungus *Simplicillium* sp. associated with soft coral *Sinularia* sp. synthesizes five new peptides, including *Sinularia* peptides A–E. These bioactive AMPs exhibit significant antimicrobial activity against *Mycobacterium tuberculosis*, *Colletotrichum asianum*, and *Pyricularia oryzae* Cav. Mollusks such as *Mytilus edulis*, *Ruditapes decussatus*, and oyster *Mytilus galloprovincialis* produce AMPs such as myticins and mytilin. A cyclic hexapeptide, cyclo-(Gly-Leu-Val-IIe-Ala-Phe), bacicyclin isolated from *Bacillus* sp. associated with *M. edulis*, exhibits antibacterial activities against clinically relevant bacterial strains such as *S. aureus* and *Enterococcus faecalis* (Wiese et al., 2018; Zanjani et al., 2018). AMPs derived from marine invertebrates can modulate the lifecycle of bacterial biofilm and also inhibit biofilm formation. Crustin, an antibacterial protein, consists of alanine or threonine, glycine, and glutamine residues at their cleavage site and is derived from the hemolymph of crustaceans (Destoumieux-Garzón et al., 2016). It effectively inhibits biofilm formation of various antibiotic-resistant bacterial strains, including *B. pumilis* and *B. thuringiensis* and also is effective against *Aeromonas hydrophila* and *E. coli* (Rekha et al., 2018; Sivakamavalli et al., 2020). A novel antibacterial peptide named PcnAMP, extracted from *Procambarus clarkia* (Pcn) (a red swamp crayfish), exhibits a significant inhibitory effect against Gram-positive and GNB strains such as *S. aureus* and *M. luteus* (Zhao B. R. et al., 2020). AMPs from ascidian *Didemnum* sp. exhibit an antibacterial effect against human pathogens *E. faecalis*, *S. marcescens*, *S. typhimurium*, and *S. aureus* at MICs of 2.30, 2.17, 2.05, and 1.95 μg/ml, respectively (Arumugam et al., 2020). The AMPs halocyntin and papillosin from tunicate *H. papillosa* exhibit antibacterial activity against *M. luteus* and *E. coli* (Palanisamy et al., 2017). A novel AMP myticusin-beta isolated from the mantle of *Mytilus coruscus* exhibits a broad range of antibacterial activity and acts as a substitute to antibiotics (Oh et al., 2020). Therefore, the diverse forms of marine invertebrates act as natural reservoirs for novel AMPs, which can be exploited for the treatment of various microbial infections (Thoms et al., 2007; Destoumieux-Garzón et al., 2016; Table 3).

**TABLE 3:** Antimicrobial peptides from marine organisms.

**Name of the peptide**	**Source of peptide**	**Mode of action**	**Inhibitory concentration**	**References**
**An antimicrobial peptide from marine invertebrates**				
Sinulariapeptides A–E	*Coral Sinularia* sp.	Inhibitory effects against protein tyrosine phosphatases of *Mycobacterium tuberculosis* (MptpA and MptpB)	IC_50_ values of 35.0 and 25.9 μM against MptpA and MptpB	Dai et al., 2018
Bacicyclin	*Mytilus edulis*	Cell membrane damage of *Enterococcus faecalis* and *Staphylococcus aureus*	MIC values of *Enterococcus faecalis* and *Staphylococcus aureus* was noted to be 8 and 12 mM, respectively	Wiese et al., 2018
Crustin	*Portunus pelagicus*	The growth reduction and biofilm inhibition potential of on Gram-positive bacteria and Gram-negative bacteria	MIC of both Gram-positive and Gram-negative bacteria was noted to be 30 and 20 μg/ml, respectively	Rekha et al., 2018
**An antimicrobial peptide from marine microorganisms**				
Hyporientalin A	*Trichoderma orientale*	Growth inhibitory effects toward clinical isolates like *Candida albicans*	MICs of *Candida albicans* species (247FN and 098 VC) was noted to be 2.55–4.92 μM, respectively	Touati et al., 2018
Fengycins	*Bacillus subtilis*	Inducing the mitochondrial membrane potential (MMP), reactive oxygen species (ROS), downregulate the ROS-scavenging enzymes and chromatin condensation in plant-pathogenic fungus *Magnaporthe grisea*		Zhang and Sun, 2018
EeCentrocin 1	*Echinus esculentus*	Cell membrane damage	MIC of *Corynebacterium glutamicum* and *S. aureus* (MIC = 0.78 μM)	Solstad et al., 2019
Tetrapeptides 1	*Streptomyces* sp.	Growth inhibition of *Burkholderia gladioli* and *Burkholderia glumae*	MIC was noted to be 0.068 and 1.1 mM in *Burkholderia gladioli* and *Burkholderia glumae*	Betancur et al., 2019
Thr-Pro-Asp-Ser -Glu-Ala-Leu (TPDSEAL)	*Porphyra yezoensis*	The surface of *S. aureus* became blurred, loose, irregular, and cell wall damage		Jiao et al., 2019
**An antimicrobial peptide from marine vertebrates**				
Epinecidin-1	*Epinephelus coioides*	Disrupted the membrane of metronidazole-resistant *Trichomonas vaginalis* and completely killed the pathogen	Minimal Epi-1 concentration was noted to be 62.5 μg/ml to produce 100% growth inhibition of *Trichomonas vaginalis*	Huang et al., 2019
Tissue factor pathway inhibitor 1 (TFPI-2)	*Sciaenops ocellatus*	TFPI-2 destroying cell membrane integrity, penetrating the cytoplasm and inducing degradation of genomic DNA and total RNA	MICs of TFPI-2 against *M. luteus*, *S. aureus*, *V. litoralis*, *V. ichthyoenteri*, *V. vulnificus*, and *V. scophthalmi* were 3, 6, 11, 85, 170, and 340 μM, respectively	He et al., 2018
Caspian trout (CtHep)	*Salmo caspius*	The growth inhibition of infectious bacteria	MICs concentration was noted to be 50 and 12.5 μM for *Aeromonas hydrophila* and *Bacillus subtilis*	Shirdel et al., 2019

### Antimicrobial Peptides From Marine Microorganisms

Marine microbial systems are the significant resources of AMPs with unique pharmacological features, including antimicrobial, cytostatic, animal growth, immunosuppressant, antiviral, antimalarial, antiparasitic, promoters, and insecticides activities (Semreen et al., 2018). AMPs extracted from symbiotic marine microorganisms exhibit enhanced broad-spectrum antimicrobial activity. These natural compounds are now being exploited to resolve the microbial drug-resistance problem. Hyporientalin A, an anti-*Candida* peptaibol, a moronecidin-like peptide from *Trichoderma orientale* strains, symbiotic fungi of Mediterranean marine sponge *Cymbaxinella damicornis*, inhibits the growth of clinical isolates of *C. albicans*, Gram-positive and Gram-negative bacteria (Touati et al., 2018). Cyclic lipopeptide Fengycins from marine bacterium *B. subtilis* (BS155) is effective against the plant-pathogenic fungus *Magnaporthe grisea*. Host-dependent marine microbes are excellent sources of many active antimicrobial cyclic peptides (e.g., the cyclolipopeptides cyclodysidins A–D). These peptides, secondary metabolites of *Streptomyces* sp. associated with sponge *Dysidea tupha*, exhibit broad-spectrum antimicrobial activities (Indraningrat et al., 2016). Different marine gamma-proteobacteria associated with seaweeds, particularly, *Pseudomonas* sp., are the primary sources in cyclotetrapeptide cyclo-(isoleucyl-prolyl-leucyl-alanyl), cyclic heptapeptide, scopularides A and B, and ogipeptin A–C. These peptides exhibit intense antimicrobial and anthelmintic activities. Ogipeptin is a powerful agent suppressing the immunostimulatory role of lipopolysaccharides present in the cell wall of GNB (Betancur et al., 2019). Similarly, the marine sponge *Tethya aurantium* associated with fungus *Scopulariopsis brevicaulis* synthesizes cyclodepsipeptides scopularides A and B that exhibit effective cytotoxic activity against pathogens (Agrawal et al., 2017). New cyclic lipopeptides maribasins A and B from the broth culture of marine microorganism *B. marinus* exhibit broad-spectrum activities against phytopathogens such as *Fusarium oxysporum*, *Fusarium graminearum*, *Verticillium alboatrum*, *Alternaria solani*, and *R. solani* with the MICs of 25–200 mg/ml (Zhang et al., 2010). Additionally, the two new cyclic tetrapeptides, from the marine strain *Streptomyces* sp., are effective against *Burkholderia gladioli* and *Burkholderia glumae* at MIC of 0.068 and 1.1 mM, respectively. Furthermore, tetrapeptide-2 is effective against *B. glumae* (MIC = 1.1 mM) and fungal phytopathogens (Betancur et al., 2019). Hence, the diversified marine microorganisms prove to be an effective substitute to the existing antibiotics, thereby reducing the probability of antibiotic-resistant pathogens (Table 3).

### Antimicrobial Peptides From Marine Vertebrates

Antimicrobial peptides in marine vertebrates are mainly localized in body fluids, mucous layers, and epithelial surfaces (Edilia Avila, 2017). AMPs participate in body defense mechanisms to eliminate the invading pathogens and enhance physiological and metabolic processes such as toxin neutralization, wound healing, angiogenesis, and iron metabolism. For instance, epinecidin-1 (Epi-1) disrupts the cell membrane of metronidazole-resistant *Trichomonas vaginalis* and terminates the pathogen with a minimal dose of 62.5 μg/ml. *T. vaginalis* treated with different concentrations of Epi-1 (62.5, 125, 250, or 500 μg/ml) exhibits 100% growth inhibition (Huang et al., 2019). 3C-terminal peptide tissue factor pathway inhibitor 1 (TFPI-1) from *Cyprinus carpio* (common carp) exhibits bactericidal effects against *M. luteus*, *S. aureus*, and *Vibrio vulnificus* (Su et al., 2020). Orange-spotted grouper (*Epinephelus coioides*) derived from AMP EPI is effective against GPB (Su and Chen, 2020). Cysteine-rich Hepcidins (CtHep) from vertebrates such as fish, reptiles, and amphibians can significantly inhibit *Streptococcus iniae* and *A. hydrophila* (Shirdel et al., 2019). Marine betta fish *Betta splendens* produce four families of AMPs, including defensins, piscidins, hepcidins, and LEAP-2, which vigorously suppress the growth of fungi, bacteria, virus, and parasites (Amparyup et al., 2020). A short novel peptide synthesized from the core region of the LCNKL2 of a marine fish *Larimichthys crocea* inhibits *S. aureus* and *Vibrio harveyi* (Zhou et al., 2019). Antibacterial activity of piscidin-5 like AMP has been reported from *L. crocea* (Pan et al., 2019). Therefore, AMPs are essential to induce adaptive response and participate in a vertebrate’s metabolic and reproductive processes (Table 3).

## Conclusion

The exponentially increasing cases of antibiotic resistance requires the introduction of novel and alternative drug molecules. Insects, nematodes, insect–nematode–bacterial associations and marine organisms could be promising sources for natural AMPs to address the challenges of multidrug-resistant infections. The conventional method of overmining natural antibiotic sources has failed to develop new drugs to overcome drug resistance. Genomic analysis indicates the presence of several gene clusters for the novel secondary metabolite biosynthesis. The exploitation of these secondary metabolites might lead to the discovery of potential antimicrobial compounds. This review thereby highlights the symbiotic bacteria–EPN complexes as prospective antimicrobial peptide sources and opens the window to new sources of intervention and invention of natural bioactive compounds to combat antimicrobial resistance. Further research is required to understand the metabolic pathways to optimize the conditions for large-scale production and commercialization of these drug molecules as adequate substitutes.

## Author Contributions

SD, FJ, and XX conceptualized the manuscript. SD, AP, and CM drafted the manuscript. AP was responsible for preparing the figures in the manuscript. FJ, NS, SD, AP, and CM assisted in revising the manuscript. All authors contributed to the article and approved the submitted version.

## Conflict of Interest

The authors declare that the research was conducted in the absence of any commercial or financial relationships that could be construed as a potential conflict of interest.

## Footnotes

1 http://aps.unmc.edu/AP/main.php/
